# An Update on Neurosurgical Management of Primary CNS Lymphoma in Immunocompetent Patients

**DOI:** 10.3389/fonc.2022.884724

**Published:** 2022-04-20

**Authors:** Florian Scheichel, Daniel Pinggera, Branko Popadic, Camillo Sherif, Franz Marhold, Christian Franz Freyschlag

**Affiliations:** ^1^ Karl Landsteiner University of Health Sciences, Krems, Austria; ^2^ Department of Neurosurgery, University Hospital St. Poelten, St. Poelten, Austria; ^3^ Department of Neurosurgery, Medical University of Innsbruck, Innsbruck, Austria

**Keywords:** Primary central nervous system lymphoma (PCNSL), corticosteroid therapy, diagnostic workup, diagnostic delay, diagnostic yield

## Abstract

Primary central nervous system lymphomas (PCNSL) are rare CNS tumors that harbor a conspicuously longer diagnostic delay compared to other malignant brain tumors. The gold standard for diagnosis is stereotactic biopsy to acquire tissue for histopathological analysis and therefore neurosurgery plays a central role when reducing the diagnostic period is mandated. However, histopathological diagnosis could be complicated if the patient was preoperatively exposed to corticosteroids. Besides the histopathological result, diagnosis of a PCNSL also requires full diagnostic workup to exclude cerebral metastatic disease of a systemic lymphoma. Most reviews of PCNSL discuss recent advancements in systemic treatment options from an (neuro-)oncologic viewpoint, whereas our intention was to discuss the optimization of the diagnostic period and therefore describe current standards of imaging, summarizing the diagnostic workup, discussing the surgical workup and future diagnostic prospects as well as the influence of preoperative corticosteroid therapy to reduce the diagnostic delay of PCNSL patients.

## Introduction

Primary central nervous system lymphoma (PCNSL) is defined as extranodal malignant non-hodgkin lymphoma of the brain, spinal cord or the leptomeninges in absence of systemic involvement. Histologically, most PCNSL are diffuse large B-cell lymphomas, followed by Burkitt, lymphoblastic, marginal zone and T-cell lymphomas (1). It therefore constitutes a fairly rare CNS neoplasm with an incidence of 0.26 to 0.48 per 100.000 person-years, which accounts for approximately 3% of all primary brain tumors (2–4). Immunocompromised patients after transplantation or being affected by AIDS have a higher relative risk of development of PCNSL (5). Recently, most PCNSL patients are immunocompetent patients and the incidence within an elderly cohort is still increasing (4, 6, 7). Clinical symptoms can be concealed with cognitive impairment being the most frequent, followed by gait disturbances, focal neurologic deficits, symptoms of increased intracranial pressure and seizures (8). Treatment for PCNSL differs from systemic lymphomas and consists of different chemotherapy regimens, all containing systemic high-dose methotrexate (9, 10). Additionally, autologous stem cell transplantation is becoming more important, whereas radiotherapy is only rarely applied, e.g. in selected cases not suitable for aggressive systemic therapy (11, 12). The median community based overall survival has increased from 8.9 to 10 months to 25.3 months in recent studies, with a 5-year survival rate of 38% (7, 8, 13). Known favorable prognostic factors are high Karnofsky performance status and younger age at the time of diagnosis and treatment initiation (8). The primary role of neurosurgery is focused on safe and efficient planning and procedure of surgical biopsy to acquire tissue for histopathological diagnosis. Diagnostic biopsy represents the most time-critical step in the further course of the disease, resulting in a timespan between onset of symptoms to histopathological diagnosis described to range between 35 and 75 days, whereas more recent studies showed a decrease in this period (8, 13, 14). As a result, the diagnostic delay from the first imaging to definitive histopathological diagnosis is found to be significantly longer in PCNSL compared to, e.g., glioblastoma (15).

Most reviews of PCNSL encompass recent advances of systemic treatment from a neuro-oncological viewpoint. The aim of this particular review was to focus on the period between imaging and diagnosis, surgical planning and potential pitfalls in diagnosis, especially after corticosteroid therapy. Furthermore, we want to provide an overview of the diagnostic workup, which should be performed until histopathological confirmation.

## Imaging

**Figure 1:**
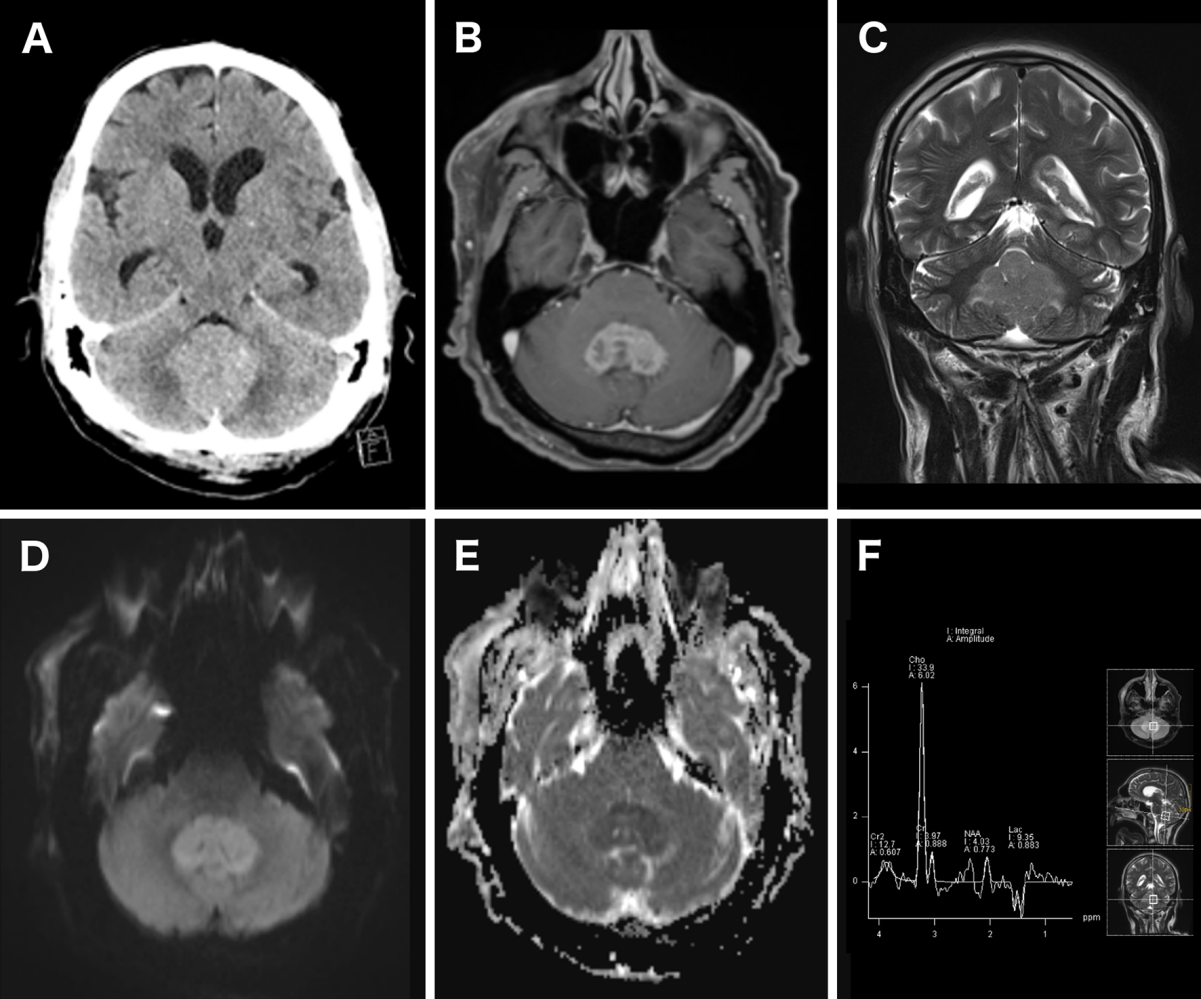
Imaging of a patient with a histopathological proven PCNSL. Unenhanced CT scan showed a hyperdense cerebellar lesion **(A)**. Contrast-enhanced T1-weighted axial MRI showed a strong and homogenous contrast enhancement of the lesion **(B)**. The lesion homogeneously appeared hyperintense in T2-weighted MRI **(C)**. Diffusion restriction was detected as well, resulting in a bright DWI (b = 1,000) **(D)** and dark ADC map signal **(E)**. 1H-MR spectroscopy showed an increased choline peak and decreased creatinine and N-acetylaspartat **(F)**.

If PCNSL is suspected, early competent imaging analysis is crucial as it strongly influences further decision making and helps avoid hasty corticosteroid treatment (CST) before surgery. In clinical practice, unenhanced computed tomography (CT) is mostly used as first imaging resource after emergence of symptoms. Sometimes the classical location and appearance in CT imaging can already be indicative of PCNSL, radiographically, as CT shows an iso- to hyperdense lesion due to the hypercellularity and the relatively high ratio of the nucleus to the cytoplasm in PCNSL (**Figure 1A**) (16, 17). Though magnetic resonance imaging (MRI) is warranted for the imaging modality of choice for diagnosis and surgical planning, its accessibility during nights and weekends could be reduced. Upon imaging, typical regions where PCNSL can be found are periventricular, in the corpus callosum and deep gray matter (9, 18). In 30–48% of all cases PCNSL show multiple lesions (13, 17, 19). Classic findings in MRI are iso- to hypointense lesions on unenhanced T1-weighted MRI and iso- to hyperintense on T2-weighted MRI sequences (**Figure 1C**) (17, 20). In immunocompetent patients usually there is a strong to moderate homogenous contrast enhancement (**Figure 1B**), but rarely atypical enhancement patterns and cases with no enhancement have been described (17–19). Advanced imaging like diffusion-weighted imaging (DWI) and spectroscopy can help distinguish the lesion from other entities in atypical cases (21). DWI is usually restricted due to high cellularity, resulting in a hyperintense signal b-1000 and hypointense signal on ADC maps (**Figures 1D, E**) (17, 21, 22). Furthermore, low ADC values where shown to be a surrogate parameter for cellular density and potentially predict outcome (23). Spectroscopy usually shows a large choline peak, decreased N-acetylaspartate (NAA), a decrease of creatine and an increase in lactate and lipids (**Figure 1F**) (17, 19). Additional information can be obtained by performing cerebral ^18^F-Fluorodeoxyglucose-positrone-emission tomography (FDG-PET). FDG-PET analysis hereby focuses mainly on standardized uptake values (SUV), which are higher in PCNSL compared to glioblastomas (24).

Recent studies on the use of radiomics, based on either MRI or FDG-PET, to differentiate PCNSL from other entities, particularly glioblastoma, have shown promising results (25–29) and wider use in practice is desirable, as quantitative imaging or the combined analysis of a radiologist and radiomics provide better diagnostic results than radiologists alone (26, 29). However, diagnostic models for PCNSL have been defined in retrospective studies with small patient cohorts and need to be validated in large data sets and in prospective multicenter studies. Solving other challenges of quantitative imaging techniques such as reproducibility, standardization, and different imaging protocols between different centers should only be a matter of time (30).

## Diagnostic Workup

The diagnostic workup of PCNSL has the aim to rule out a systemic involvement and should either be performed while waiting for surgery or for the histopathological result. The preclusion of systemic disease is important as it influences the treatment regime and therefore time to initiate chemotherapy can be shortened. An interdisciplinary effort of neurology, radiology, neurosurgery, neuropathology and medical oncology is necessary to complete the accurate diagnosis of a PCNSL and to determine the extent and degree of the disease.

If spinal symptoms are suspected, MRI of the complete neuroaxis should be performed to rule out spinal or meningeal involvement (31).

Independent of visual symptoms, ocular examination including fundoscopy should be performed to determine potential ocular involvement (9), which can be found in 15 to 25% of all patients and needs further ophthalmological therapy (32).

Furthermore, staging should contain at least a contrast-enhanced CT scan of the chest, abdomen and pelvis, testicular ultrasound in older men and bone marrow biopsy (9, 31).

In patients without systemic involvement in contrast-enhanced CT, ^18^F-Fluorodeoxyglucose-PET revealed systemic PCNSL in 8% (33, 34) but it is not as easily available as contrast-enhanced CT and therefore not performed on a regular base.

Lumbar puncture should be performed after the preclusion of contraindication in imaging as CSF cytomorphology and flow cytometric analysis potentially allow a definitive diagnosis and can in some cases obviate the need for surgery (9). However, the diagnostic yield of cytomorphologic lumbar puncture is only 6–13.3%, which inevitably requires biopsy in most cases (35–38). Only if lumbar puncture successfully acquires the diagnosis, the inherent risk of brain biopsy can be avoided. However, prolonged time to therapy could decrease the outcome in PCNSL (15), and thus lumbar puncture should not delay surgery in clinical practice. As the period from imaging to histopathological diagnosis has been described to be as long as 28 days (14) and the time from imaging to biopsy 19 days (15), LP and CSF analysis could be performed without delay of surgery in many cases if performed early in the clinical course. Notably, recent research on additional analyses in liquid biopsy of CSF and serum showed promising results harboring great potential to possibly replace diagnostic brain biopsy in PCNSL. CSF analysis for CD79B and MYD88 or diagnostic markers like CXCL-13, B2M, and neopterin are promising prospects, yet there is currently not enough evidence for standardized clinical use (39–41) and therefore brain biopsy remains the current gold standard for diagnosis. However, digital PCR of cell-free DNA for mutations in the MYD88 gene showed a sensitivity and specificity of up to 100% in a small series by Yamagishi et al. (42). Moreover, the detection of mutations in genes such as MYD88 or CD79B in liquid biopsy could have additional clinical implications, as these mutations could enable targeted therapies (43). Because liquid biopsy has the advantage of being minimally invasive and does not require scheduling, it could also help reduce diagnostic delays once the findings allow for broad clinical application. The impact of CST on the diagnostic accuracy of liquid biopsy has not yet been studied.

## Surgical Workup

**Figure 2:**
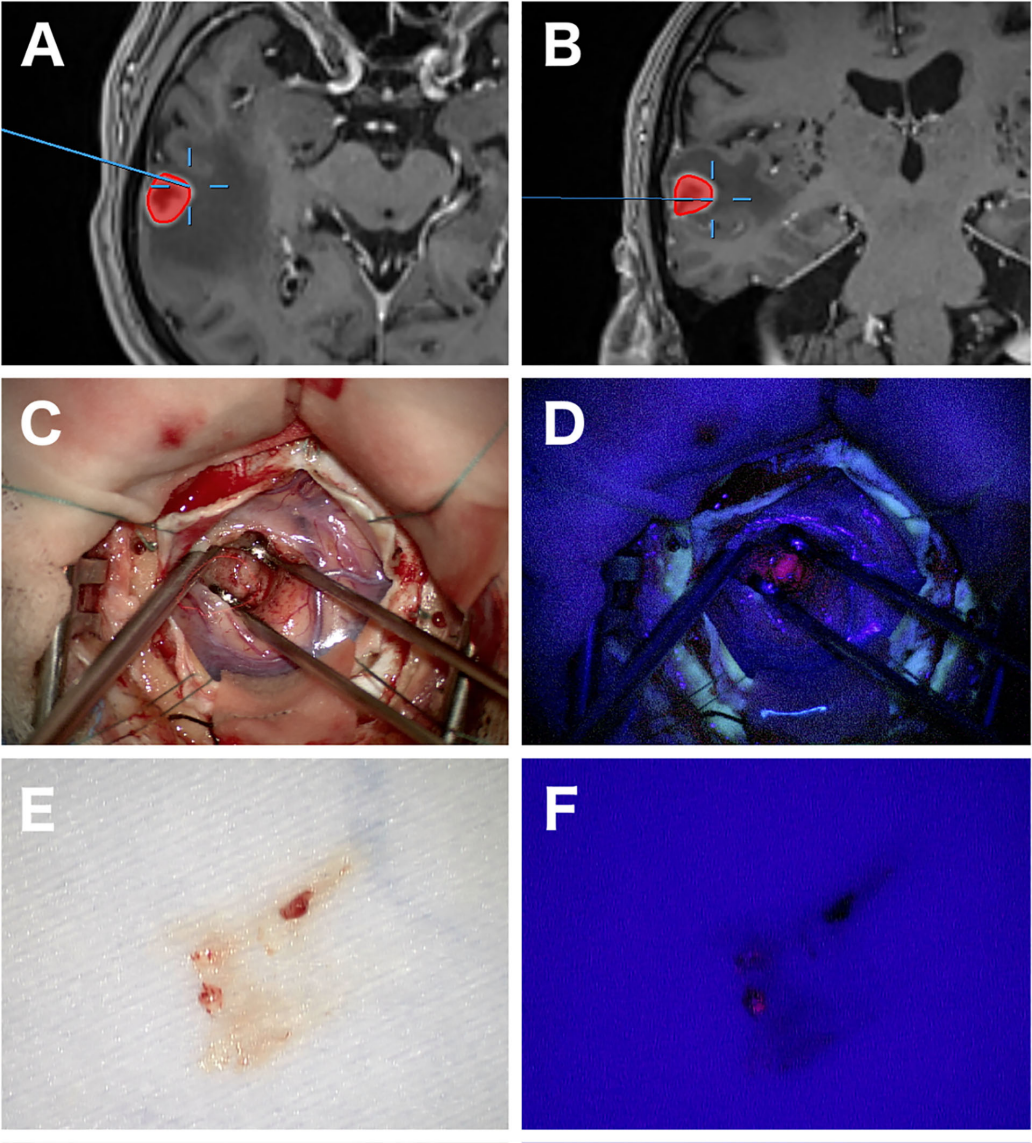
Images of open biopsy of a PCNSL with the aid of 5-ALA fluorescence. Axial and coronal navigational MRI showing a heterogeneous contrast enhancing lesion in the right temporal lobe and the exact location of the biopsy **(A, B)**. Intraoperative images at the biopsy location with strong 5-ALA fluorescence **(C, D)**. A tissue specimen later diagnosed as PCNSL showing positive fluorescence under 405 nm wavelength blue light in another patient **(E, F)**.

Stereotactic or frameless biopsy is the standard neurosurgical procedure for acquiring tissue in PCNSL and achieves a diagnostic yield of more than 91% (13, 44). Overall, stereotactic biopsy is accompanied a periprocedural morbidity of 8.5% and mortality of 0.9% (45). Additional periprocedural techniques like frozen section (46) and 5-ALA fluorescence (47, 48) might help determine whether diagnostic tissue has been acquired (**Figure 2**). 5-ALA-Fluorescence in PCNSL is described in 79–83% with a high positive predictive value for diagnostic tissue (47, 49). Furthermore, positive 5-ALA fluorescence can help shorten surgical duration (50). Open surgery was historically without significance in PCNSL patients due to worse outcomes in older studies (51–53). However, this tenet has been challenged in a recent study by Weller et al. (54). The authors described an improvement of progression-free survival (PFS) and overall survival (OS) after resection compared to biopsy in their *post hoc* analysis. Yet, patients with single lesions more often underwent resection, and after further statistical adjustment for the number of lesions, only advantage for PFS remained. Other studies came to a similar conclusion reflecting that this might be due to a selection bias for patients with single lesions and patients without the involvement of deep structures (55–57). In contrast, a large retrospective study by Houillier et al. including 1002 patients did not find a difference in outcome regarding the type of surgery (8). As surgical procedures evolved, surgical resection appears to be safe nowadays in selected cases, but its clinical significance still must be determined in further studies (58).

## Preoperative Corticosteroid Therapy

Preoperative corticosteroid therapy in PCSNL has been a point of debate for many years. As PCNSL cells may react with cell arrest and apoptosis to corticosteroid therapy (59–61), transient tumor shrinkage and morphological changes can be seen in up to 50%, potentially hindering histopathological diagnosis (62, 63). This phenomenon also gave PCNSL the name “ghost” or “vanishing” tumor (64). This was formerly described as diagnostic for PCNSL, but is obsolete nowadays, as also other tumor entities were identified to show transient regression after CST (65–68). Retrospective studies showed an increased rate of inconclusive biopsies after CST of 11–22% (13, 14, 69), while recent retrospective studies showed that there is not necessarily a decrease in the diagnostic yield after preoperative CST (44, 70–72). However, besides a potential selection bias, these studies lacked the statistical power to identify small differences in diagnostic rates. A recent combined analysis of the available studies showed an odds ratio of 3.3 for inconclusive biopsy after CST (44). Although absolute numbers of inconclusive biopsies decreased, the odds ratio for inconclusive biopsy after preoperative CST remained the same. Thus, CST should not be administered before surgery if PCNSL is suspected and tissue must be acquired (9). Yet, the clinical condition of patients sometimes require preoperative CST treatment, resulting in most PCNSL patients receiving CST preoperatively (13). A single dose of CST can already pose a challenge for histopathological diagnosis in some cases but prolonged CST led to a higher incidence of inconclusive biopsies in one study (69), therefore accurate evaluation of duration and dosage of CST is mandatory for further workup. The optimal management of PCNSL patients with preoperative CST remains controversial. The exact time of CST tapering that is necessary to overcome the influence of CST on diagnostic yield is not defined (13, 44). In practice, if a contrast-enhanced lesion shows distinct regression, surgery is usually delayed until new progression is evident in serial MRI (9, 73). In case of a PCNSL that only reacts with little or no regression, the risk of inconclusive biopsy must be weighed up against the significant delay to definitive therapy when biopsy is delayed.

## Discussion

Diagnosis and therapy of PCNSL is a multidisciplinary task, with brain biopsy as performed by neurosurgery being at the center of it. These multiple intersections between different disciplines like neurology, radiology, neurosurgery, pathology and oncology harbor a risk of unnecessary delays and might account for the prolonged diagnostic period of PCNSL compared to other brain malignancies (15). At present, no clear evidence has been found that resection offers an outcome advantage for the patient. Therefore, and contrary to many other brain malignancies, neurosurgery cannot influence the outcome of the patient with resection itself. This highlights the potential benefit of non- or minimally-invasive diagnostic tools that have lower morbidity than surgery. Liquid biopsy, alone or along with quantitative imaging techniques, has great potential to replace stereotactic biopsy in diagnosis of PCNSL especially in radiologically typical cases. Although the available data do not allow a standard application, Yamagishi et al. described a case of pontine PCNSL that was successfully treated on the basis of diagnosis by imaging and MYD88 mutation analysis in CSF (42). In such cases where brain biopsy is expected to cause high morbidity and when PCSNL is highly suspected, diagnosis by liquid biopsy should be considered. However, based on the available evidence, brain biopsy remains the current gold standard and the goal for neurosurgery must be a most efficient management and safe diagnostic brain biopsy to facilitate adjuvant treatment. Even if biopsy is performed early in the clinical course, there is still a median period of 14 days to definitive adjuvant treatment (7). This time needs to be used efficiently to avoid further delay of definitive treatment and its potential negative influence on the outcome. A major clinical issue that may be responsible for distinct diagnostic delay is preoperative CST, especially if CST must be stopped before surgery due to regression (13). If clinically possible, CST should therefore strictly be avoided in potential PCNSL patients as it increases the rate of inconclusive biopsies (44). In patients with no history of malignancy or immunosuppression and periventricular tumors upon initial CT, we recommend withholding the patient from CST treatment until MRI provides further diagnostic information and subsequent steps of diagnosis should be executed shortly after. It remains unclear how long the pause of CST treatment should be carried out. Currently, many centers wait for another progression of PCNSL upon MRI, which leads to a significant delay. The ongoing debate on the clinical impact of preoperative CST must be clarified through future prospective studies. However, current evidence shows that the risk of inconclusive biopsies is significantly higher with preoperative CST treatment (44, 62). Even though absolute rates of inconclusive biopsies after CST are quite low in recent studies, it is recommendable to avoid this issue in the first place if possible.

**Figure 3:**
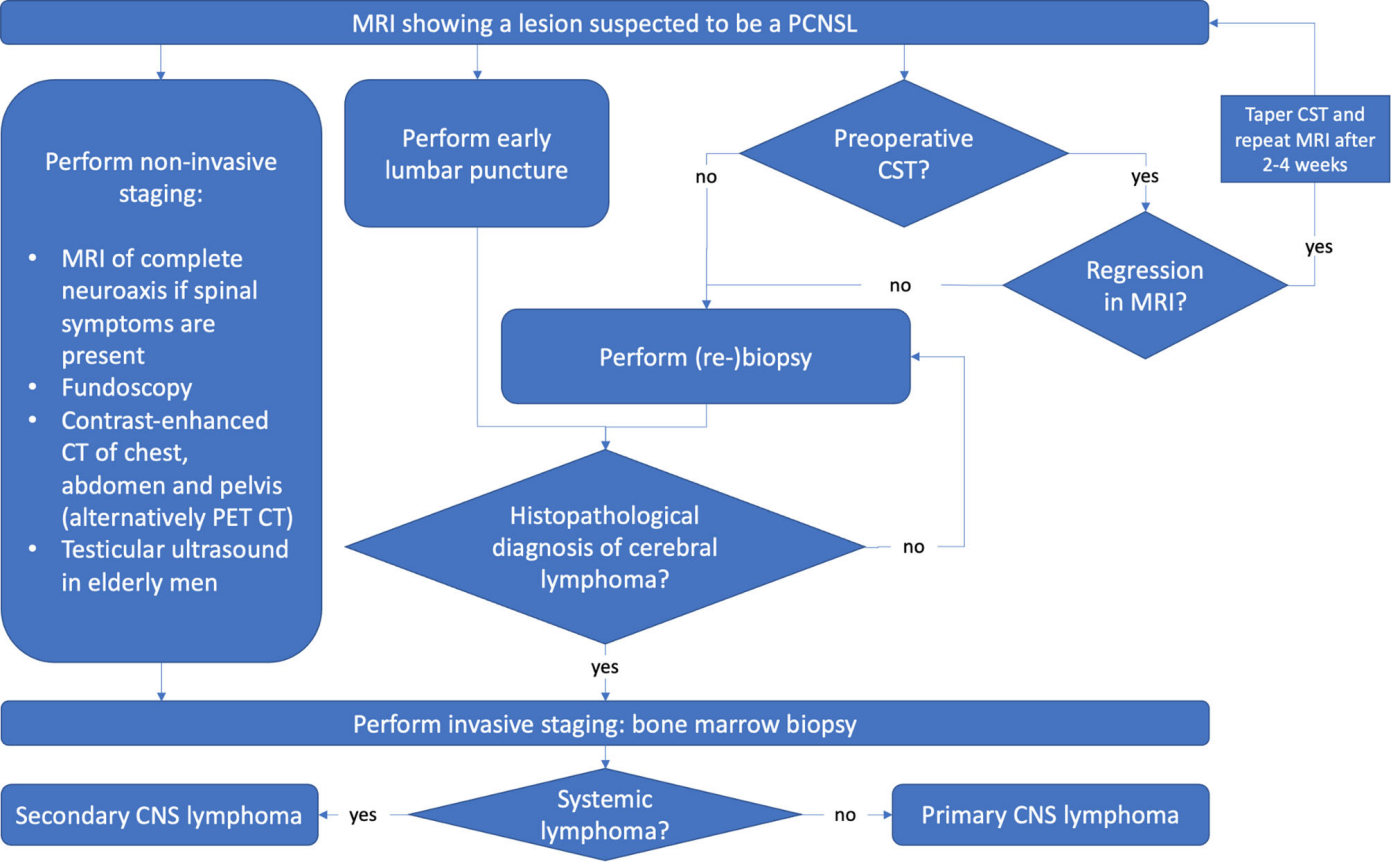
A systematic workflow for diagnosis of PCNSL. Ideally, lumbar puncture and CSF analysis should be performed early without delaying biopsy. Non-invasive staging should be performed while waiting for biopsy or histopathological results to reduce diagnostic delay.

To sum up, a potential PCNSL must be recognized as early as possible to avoid preoperative CST and schedule early surgery. Next, full diagnostic workup of PCNSL should be initiated while waiting on surgery or histopathological results to reduce delay in therapy (**Figure 3**).

## Author Contributions

FS, DP, BP, FM, and CF contributed to conception and design of the study. FS wrote the first draft of the manuscript. All authors listed have made a substantial, direct, and intellectual contribution to the work and approved it for publication.

## Funding

The article processing charge was covered by the Open Access Publishing Fund of Karl Landsteiner University of Health Sciences, Krems, Austria.

## Conflict of Interest

The authors declare that the research was conducted in the absence of any commercial or financial relationships that could be construed as a potential conflict of interest.

## Publisher’s Note

All claims expressed in this article are solely those of the authors and do not necessarily represent those of their affiliated organizations, or those of the publisher, the editors and the reviewers. Any product that may be evaluated in this article, or claim that may be made by its manufacturer, is not guaranteed or endorsed by the publisher.
